# Passive leg-lifting in heart failure patients predicts exercise-induced rise in left ventricular filling pressures

**DOI:** 10.1007/s00392-019-01531-w

**Published:** 2019-07-31

**Authors:** E. Tossavainen, G. Wikström, M. Y. Henein, M. Lundqvist, U. Wiklund, P. Lindqvist

**Affiliations:** 1 Department of Public Health and Clinical Medicine, Cardiology, Umeå University, S-90185 Umeå, Sweden; 2 Department of Medical Sciences,Cardiology, Uppsala University, Uppsala, Sweden; 3 Department of Radiation Sciences, Biomedical Engineering, Umeå University, Umeå, Sweden; 4 Department of Surgical and Perioperative Sciences, Clinical Physiology, Umeå University, Umeå, Sweden

**Keywords:** Passive leg-lifting, Exercise, Elevated filling pressures, Pulmonary hypertension, Heart failure

## Abstract

**Aim:**

The aim of this study was to assess PCWP with passive leg-lifting (PLL) and exercise, in two groups of patients presenting with normal left ventricular ejection fraction (LVEF); one group with elevated NT-proBNP (eBNP), and one with normal NT-proBNP (nBNP) plasma concentration.

**Methods and results:**

Fifty-one patients with eBNP (NT-proBNP ≥ 125 ng/l) and LVEF > 50%, were investigated and compared with 34 patients with nBNP (NT-proBNP < 125 ng/l) and LVEF > 50%. Both groups underwent right heart catheterization (RHC) at rest, PLL and exercise. From RHC, mean pulmonary arterial pressure (mPAP), cardiac output (CO), and PCWP were measured. All nBNP patients had PCWP < 15 mmHg at rest, and a PCWP of < 25 mmHg with PLL and during exercise. Patients with eBNP had higher (*p* < 0.01) resting mPAP, PCWP, and mPAP/CO. These values increased with exercise; however, CO increased less in comparison with nBNP patients (*p* = 0.001). 20% of patients with eBNP had a PCWP > 15 mmHg at rest, this percentage increased to 47% with PLL and 41% had a PCWP > 25 mmHg during exercise. Of those with PCWP > 25 mmHg during exercise, 91% had a PCWP > 15 mmHg with PLL. A PCWP > 15 mmHg on PLL had a 91% sensitivity and 92% specificity in predicting exercise-induced PCWP of > 25 mmHg.

**Conclusion:**

In patients presenting with eBNP, PLL can predict which patients will develop elevated PCWP with exercise. These findings highlight the role of stress assessment.

**Graphic abstract:**

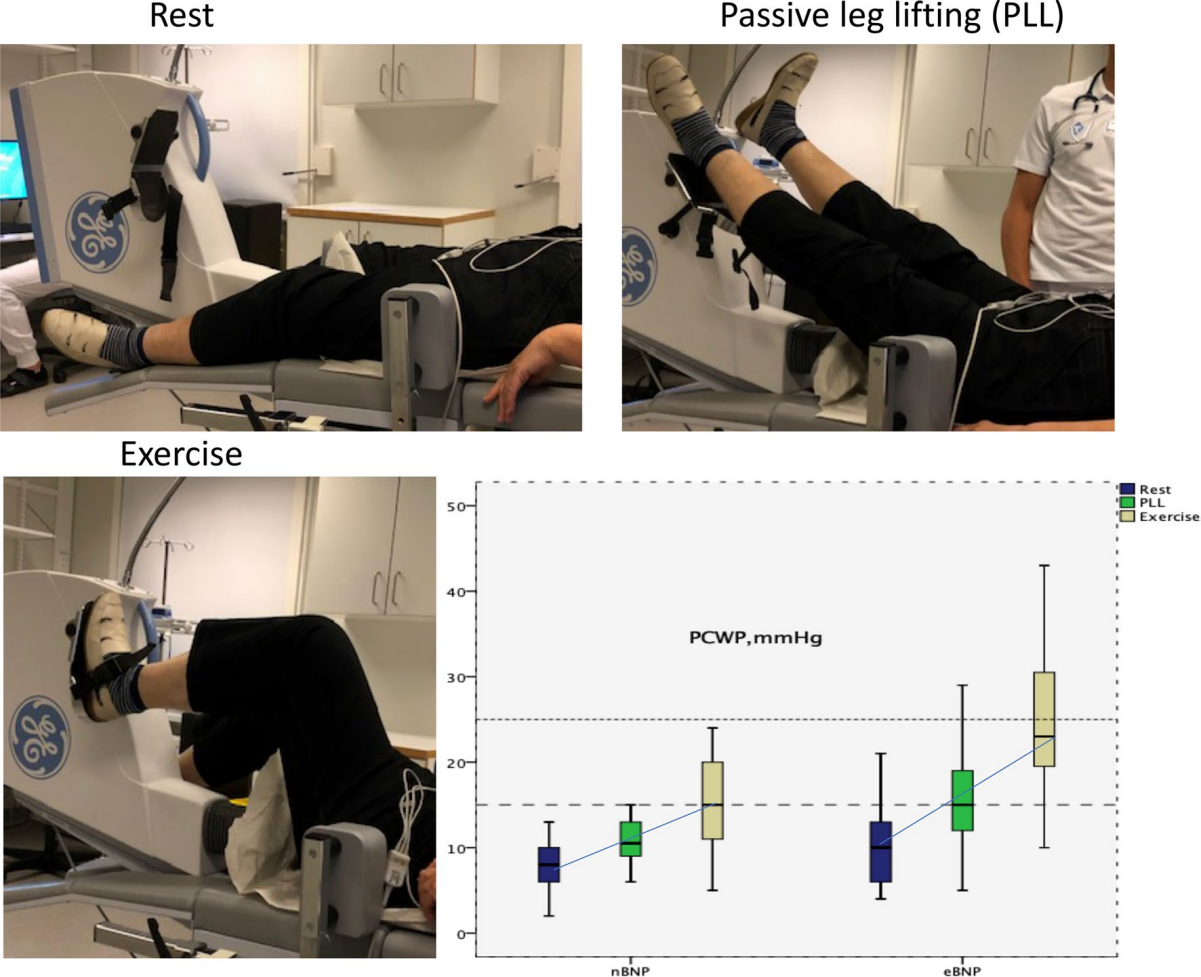

**Electronic supplementary material:**

The online version of this article (10.1007/s00392-019-01531-w) contains supplementary material, which is available to authorized users.

## Introduction

Determining the cause of dyspnea is not always clinically achievable [1]. Previously, right heart catheterisation (RHC) has been used to confirm a diagnosis, but now is commonly replaced by echocardiography [2, 3]. Echocardiography, however, has its limits regarding the estimation of left ventricular (LV) filling pressures. Another clinical dilemma is that a considerable number of patients remain limited by unexplained exertional dyspnea; despite normal estimated LV filling pressures (LVFP) at rest [4–6].

Recent studies have endorsed the use of invasive exercise hemodynamic examinations to accurately assess LV compliance in patients with inconclusive resting-RHC which remains limited by exertional dyspnea [7]. Passive leg-lifting (PLL) during RHC can identify patients with a “stiff” LV who develop raised LVFPs, venous hypertension and dyspnea on exertion [8–11]. However, the exact relationship between PLLs and LVFPs during exercise remains unknown and further investigation is required.

The aim of this study was to evaluate the use of PLL and recumbent bicycle exercise in assessing changes in LVFPs, measured by conventional invasive techniques (RHC). A group of patients with signs of HF based on elevated NT-proBNP with a normal LVEF (eBNP) are compared with patients with normal NT-proBNP and LVEF (nBNP).

## Material and **methods **

Data were collected retrospectively from patients with various clinical indications for RHC, assessed at, and registered in Uppsala University Hospital’s clinical cardiac catheterisation database. Advanced valvular disease, heart transplantation, and incomplete RHC, were considered as exclusion criteria. All patients between 2008 and 2015 were investigated for suspected HF. From this database, 153 patients were identified, 51 of those (mean age 65 ± 10 years, 24 females) had LVEF > 50% and NT-proBNP > 125 ng/ml: these patients were classified as eBNP. In addition, 34 patients (mean age 50 ± 14 years, 18 females) with LVEF > 50% and NT-proBNP ≤ 125 ng/ml, were used as a comparison and defined as nBNP patients. 57 patients with LVEF < 50%, and 11 patients who fulfilled PAH criteria were excluded. This study complied with the Declaration of Helsinki and was approved by the Ethical approval board in Uppsala.

### Right heart catheterisation (RHC)

Venous access was established by inserting a cannula in the right internal jugular vein, a median cubital vein or in the right femoral vein. In a few patients, access was not possible from the jugular vein, and those patients were catheterised from the femoral vein. A small introducer (5F) was used and no complications were observed in patients performing PLL and supine bicycle exercise.

A retrograde catheterisation was then performed using a Swan-Ganz® Standard Thermodilution Catheter (Edwards Lifesciences) [12]. Mean right atrial pressure (RAP), systolic and end-diastolic right ventricular pressures, pulmonary artery systolic, mean and diastolic pressures (PASP, PAMP, and PADP, respectively), and PCWP were all measured according to standard guidelines [13]. PCWP was measured by averaging ten consecutive pressure curve tracings.

Blood samples for estimation of oxygen saturation were drawn from the superior cava (SVC), PA and femoral artery; 8% was considered as significant oxygen saturation differential between the SVC and the pulmonary artery and thus considered to indicate a shunt. Cardiac output (CO) was determined by thermodilution [14]. Pulmonary vascular resistance (PVR) was calculated using the equation PAMP–PCWP (trans-pulmonary gradient) divided by CO. After acquiring a complete baseline RHC examination the PLL test was performed. The patient’s legs were passively lifted and rested on the bicycle pedals for up to 3 min before the start of exercise. Supine submaximal exercise testing was performed by a 6-min steady state supine bicycle exercise test with load level of 20–70 Watts depending on patient’s tolerability. PA-pressures, stroke volume and PCWP as well as calculated trans-pulmonary gradient (TPG), PVR and CO were measured at rest and again at peak exercise. PA-pressures and PCWP were also measured during PLL, and TPG was calculated.

### Statistical analysis

The statistical software package IBM SPSS Statistics, version 24 (IBM Corp. Armonk, NY, USA) and MATLAB (Mathworks Inc, Natick, MN, USA) were used for all calculations and graphic presentations. Patient characteristics were expressed as median ± inter quartile range (IQR). Group comparisons were performed using Mann–Whitney *U*-test, Cochran–Mantel–Haenszel test and Fisher’s exact probability or *X*^2^ test. Wilcoxon signed-ranks test was utilized to identify significant differences at different conditions. Two-way analysis of variance (ANOVA) was used to compare exercise data (mPAP/CO at exercise and the change from rest) from different groups, categorized into three groups after NT-proBNP and PCWP, as well as in two groups after PVR, with paired *t* tests as post-hoc tests. Sensitivity and specificity were calculated to predict elevated PCWP from resting NT-proBNP. Pearson correlation analysis was performed to evaluate relationship between NT-proBNP and mPAP and PCWP at rest, passive leg-lifting and exercise. A *p* value < 0.05 was determined as statistically significant.

## Results

Baseline characteristics are shown in Table 1.

**Table 1:** Baseline characteristics for groups classified according to normal (nBNP) or elevated (eBNP) NT-proBNP

	nBNP(34)	eBNP (51)	p-Value
Resting data			
Age, years	51 (35–63)	67 (60–71)	< 0.001
Female	18	24	0.60
ACE inhibitors	13	36	0.004
Betablockers	7	30	0.001
Diuretics	3	21	0.003
Calcium channel blockers	7	14	0.47
Atrial fibrillation or flutter	2	17	0.008
IHD	1	9	0.07
SHT	11	26	0.06
DM	1	8	0.09
NT-pro BNP, ng/L	54 (33–73)	440 (178–1402)	< 0.001
Creatinine, micro mol/L	73 (61–86)	84 (68–111)	0.015
GFR, ml/min	95 (61–86)	58 (43–76)	< 0.001
Systolic blood pressure, mmHg	126 (119–140)	130 (120–150)	< 0.045
Workload, Watt	50 (50–75)	43 (39–50)	< 0.001
Heart rate, rest, bpm	66 (59–80)	71 (58–76)	0.716
Height, cm	174 (167–179)	174 (165–180)	0.757
Weight, kg	79 (71–87)	84 (75–92)	0.125

Mann–Whitney and Cochran–Mantel–Haenszel tests were used in the analysis of background characteristics. Out of the 21 eBNP-patients with diuretics, one received Thiazide treatment instead of loop diuretics and one was treated with both Thiazide and Loop diuretics

*IHD* ischemic heart disease, *SHT* systemic hypertension, *DM *diabetes mellitus, *BNP *brain natriuretic peptides, *GFR *glomerular filtration rate

**Fig. 1:**
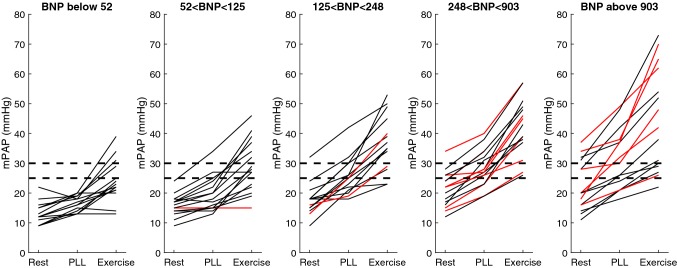
mPAP at rest, passive leg-lifting (*PLL*) and where patients are divided in quantiles based on NT-proBNP. Red lines show patients with atrial fibrillation (*AF*). Dotted lines represent 25 and 30 mmHg as cutoff values for normal values at rest and exercise

**Fig. 2:**
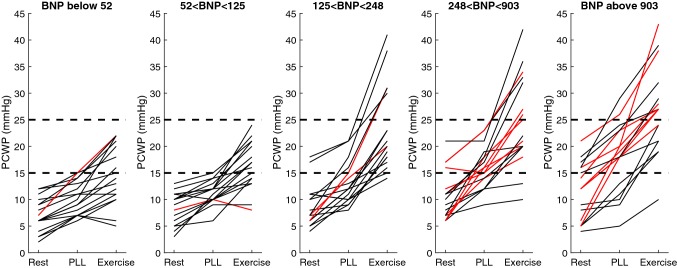
PCWP at rest, passive leg-lifting (*PLL*) and where patients are divided in quantiles based on NT-proBNP. Red lines show patients with atrial fibrillation (*AF*). Dotted lines represent 15 and 25 mmHg as cutoff values at rest and exercise

None of the patients with normal NT–proBNP had mPAP > 25 mmHg at rest, whereas one had mPAP > 30 mmHg with PLL and 8 had mPAP > 30 mmHg during exercise (Fig. 1). Also, none of the patients with normal NT–proBNP had PCWP > 15 mmHg at rest or with PLL, and none had PCWP > 25 mmHg during exercise (Fig. 2). In the same group, mPAP and PCWP increased with PLL (*p* < 0.001 for both), as did mPAP, PCWP, TPG and CO (*p* < 0.001 for all), whereas mPAP/CO slightly decreased with exercise (Table 2).

**Table 2:** Groups classified according to normal or elevated NT-proBNP

	nBNP(34)	eBNP (51)	*p* value
Resting data RHC			
mPAP, mmHg	14.5 (12–17)	18 (15–24)	< 0.001
CO, l/min	5.4 (4.7–6.0)	5.8 (4.6–7.2)	0.72
PCWP/CO, mmHg/l/min	1.4 (1.0–1.8)	1.7 (1.0–2.4)	0.09
mPAP/CO, mmHg/l/min	2.5 (2.2–3.3)	3.3 (2.6–4.3)	0.003
PCWP, mmHg	8.0 (5.8–10.3)	10.0 (6–14)	0.03
PVR, WU	1.2 (0.9–1.8)	1.6 (1.1–2.4)	0.01
TPG, mmHg	6.5 (5.0–9.3)	10.0 (7–12)	0.001
PLL data RHC			
mPAP, mmHG	17.5 (15–20)**	26 (22.5–33)**	< 0.001
PCWP, mmHg	10.5 (9–13)**	15 (12–19)**	< 0.001
TPG, mmHg	7 (5–9)	12 (9.5–16)**	< 0.001
Exercise data RHC			
mPAP, mmHg	26.5 (21–34)**	38.5 (29–49)**	< 0.001
CO, l/min	11.1 (9.8–13.0)**	9.6 (8.7–11.2)**	0.002
PCWP/CO, mmHg/l/min	1.4 (0.9–1.8)	2.4 (1.8–3.4)**	< 0.001
mPAP/CO, Hg/ml/min	2.2 (1.8–3.1)*	3.9 (3.2–5.5)**	< 0.001
PCWP, mmHg	15.0 (11–20.3)**	23 (19–31)**	< 0.001
PVR, WU	1.0 (0.6–1.3)	1.5 (1.0–2.2)	0.005
TPG, mmHg	11.5 (7.0–15.3)**	14 (9–20)**	0.10

*PLL *passive leg-lifting, *mPAP * mean pulmonary artery pressures, *CO *cardiac output, *PCWP *pulmonary capillary wedge pressures, *PVR* pulmonary vascular resistance, *TPG* transpulmonary gradient

^*^p < 0.05 compared with rest, **p < 0.001 compared with rest. Mann–Whitney test was used in the analysis between groups and Wilcoxon signed-ranks test was utilized to identify significant differences at different conditions

In patients with elevated NT-proBNP, mPAP (*p* < 0.001), PCWP/CO (*p* = 0.03), mPAP/CO (*p* = 0.002), PCWP (*p* = 0.006), PVR (*p* = 0.007), and TPG (*p *= 0.001) were all already higher than in those with normal NT-proBNP at rest. CO did not differ from patients with normal NT-proBNP at rest. In the eBNP group, mPAP (*p* < 0.001), PCWP (*p* < 0.001) and TPG (*p* < 0.05) increased with PLL. Similarly, mPAP, PCWP, and TPG (*p* < 0.001) as well as CO (*p* < 0.001) all increased with exercise in comparison to rest. PVR did not significantly change in any of the groups, however, mPAP/CO and PCWP/CO increased (*p* < 0.001) in subjects with eBNP, whereas nBNP was associated with decreased or unchanged ratios (*p* < 0.05) (Table 2). mPAP at rest, with PLL and during exercise are shown in Fig. 1. Only 20% of patients with elevated NT-proBNP had a PCWP > 15 mmHg at rest, this percentage increased to 47% with PLL and 41% had a PCWP > 25 mmHg during exercise. Out of those with elevated NT-proBNP and PCWP > 25 mmHg during exercise, 91% had PCWP > 15 mmHg with PLL (Fig. 2). The sensitivity and specificity of PCWP > 15 mmHg with PLL in predicting PCWP of > 25 mmHg during exercise were 91% and 92%, respectively. The basic characteristics and hemodynamics in patients with: (1) normal PCWP at rest and exercise; (2) normal PCWP at rest but elevated during exercise; and (3) increased PCWP at both rest and exercise are shown in Supplementary Materials, Tables 3 and 4.

**Fig. 3:**
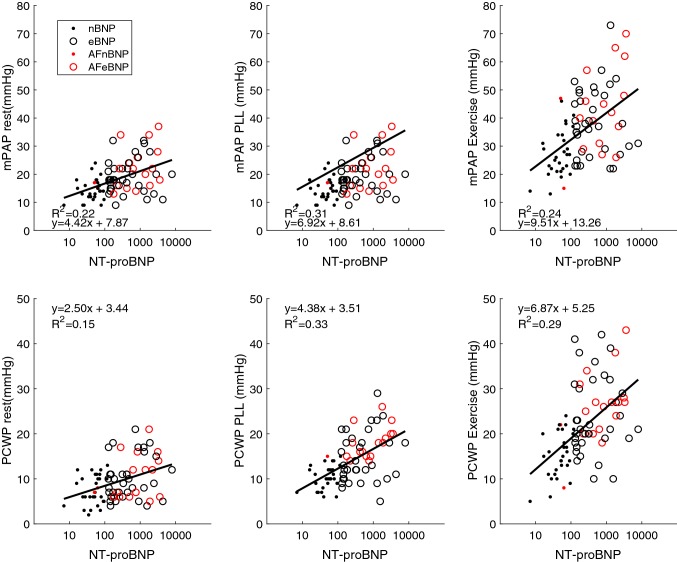
Correlation between NT-proBNP and mPAP (top) and PCWP (bottom) at rest, passive leg-lifting (*PLL*) and exercise. *nBNP* normal NT-proBNP, *eBNP* elevated NT-proBNP, *AF* atrial fibrillation

**Fig. 4:**
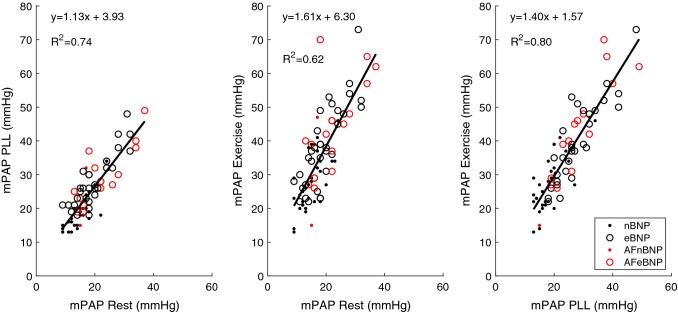
Correlation between mPAP at rest, passive leg-lifting (*PLL*) and exercise. *nBNP* normal NT-proBNP, *eBNP* elevated NT-proBNP, *AF*  atrial fibrillation

**Fig. 5:**
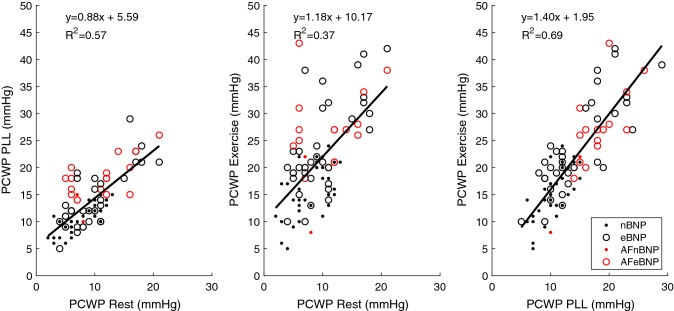
Correlation between PCWP at rest, passive leg-lifting (*PLL*) and exercise. *nBNP *normal NT-proBNP, *eBNP* elevated NT-proBNP, *AF* atrial fibrillation

In addition to significant PCWP changes during PLL and exercise, mPAP/CO and PCWP/CO both increased significantly in eBNP patients who developed high filling pressure during exercise, in contrast to a tendency towards a decrease, among nBNP patients (Table 2). The correlation between the two ratios was strong (r^2^ = 0.64). We found moderate correlations (r^2^ = 0.15–0.33) between NT-proBNP and mPAP as well as PCWP in patients with normal and elevated NT-proBNP, with or without atrial fibrillations. (Fig. 3) Furthermore, we found a strong correlation (r^2^ = 0.37–0.80) between mPAP at rest to mPAP at PLL and exercise as well as PCWP at rest and PCWP during PLL and exercise in patients with normal and elevated NT-proBNP, with or without atrial fibrillations. (Figs. 4, 5).

**Fig. 6:**
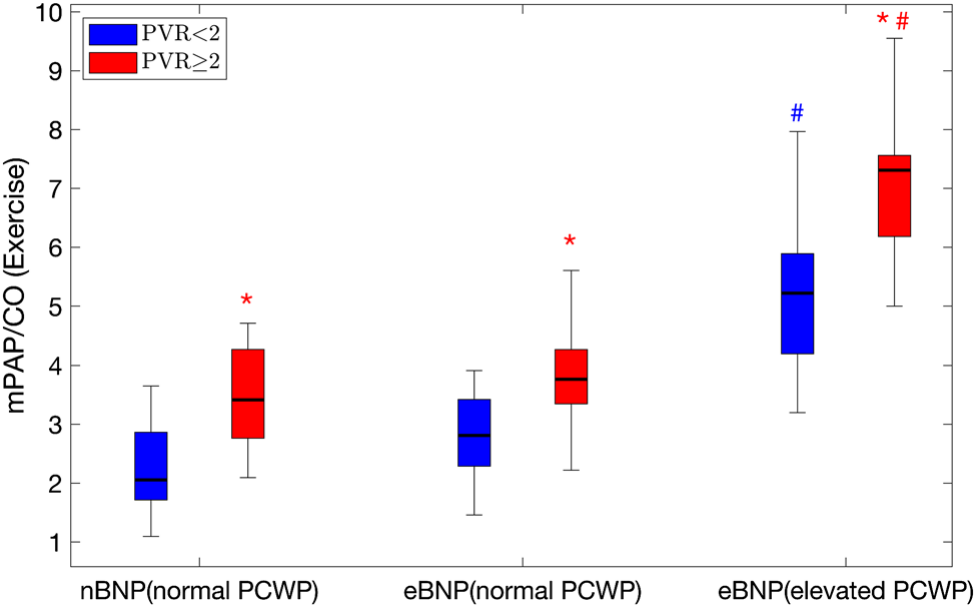
mPAP/CO (mmHg/L/min) at exercise in nBNP (left), eBNP with normal PCWP at exercise (middle), and eBNP with elevated PCWP at exercise (right). Right red boxes are patients with PVR ≥ 2WU and left blue PVR < 2WU. Note that both PVR and PCWP increased the mPAP/CO ratio. *p < 0.05 between PVR groups, within the same BNP and PCWP groups. #*p* < 0.05 elevated PCWP vs normal PCWP, within the same PVR groups. Boxes show median and IQR, whiskers show range

The ratio of mPAP/CO was also found to be associated with pulmonary vascular remodeling (Fig. 6), where there were statistically significant differences in mPAP/CO when patients were categorized based on NT-proBNP, and PWCP (ANOVA, *p* < 0.001), and PVR > 2 WU was consistently associated with a higher ratio in all three groups based on NT-proBNP and PCWP (ANOVA, *p* < 0.001). Post-hoc testing showed that mPAP/CO was significantly increased in eBNP patients who developed high filling pressure during exercise. Finally, the relationship to PVR was lost with the delta change in mPAP/CO (ANOVA, *p* = 0.83), whereas the differences between different levels of delta mPAP/CO remained (ANOVA, p < 0.001) (Supplememental files, Fig. 7). The sensitivity for NT-proBNP > 125 ng/l in predicting elevated PCWP during rest, PLL or exercise were 100% for all. The specificity for NT-proBNP > 125 ng/l in predicting elevated PCWP during rest, PLL or exercise was 46%, 57%, and 54%, respectively.

## Discussion

As a group, eBNP patients had relatively normal resting PCWP (80% of patients) at rest, which increased significantly with PLL, and further increased during exercise when compared to controls. In individual patients, only 20% had PCWP > 15 mmHg at rest; however, this increased to 47% with PLL. 41% of patients had PCWP > 25 mmHg during exercise, and 91% of them had PCWP > 15 mmHg with PLL.

This resulted in a PCWP > 15 mmHg with PLL 91% sensitive and 92% specific in predicting PCWP > 25 mmHg during exercise. In addition, an increase in delta-mPAP/CO was associated with increase in PCWP, irrespectively of PVR. Finally, elevated NT-proBNP at rest was found to be a modestly accurate predictor of elevated PCWP with PLL and during supine bicycle exercise. However, NT-proBNP within normal ranges had a strongly negative predictive value for estimating PCWP during exercise, and is strongly suggestive of normal diastolic LV compliance.

This information might be of value in daily clinical practice improving assessment and interpretation of LVFPs. The exact use of NT-proBNP in HF patients, especially with LVEF > 50%, is not well defined and varies in different studies [15, 16], however, it has been proven to be related to LVFPs and diastolic function, as well as having a good prognostic value [17].

### Data interpretation

The most common pathology in HF patients, with normal or preserved LVEF is long-standing hypertension and its consequential effects on the LV, in the form of cavity stiffness and raised filling pressures, reflected as a rise in PCWP. The majority of our eBNP patients who were limited by breathlessness did not have raised PCWP at rest; however, 41% of them developed abnormally raised PCWP > 25 mmHg during moderate work load exercise. This finding supports the diagnosis of HF and also explains patient’s symptoms. The mechanism behind normal filling pressures, despite a stiff myocardium and elevated BNP, may be due to at least two reasons. First, a large proportion of patients was already on diuretics treatment, and second, endogenous BNP secretion has a diuretic effect.

The important finding in our results was the patient response to PLL, during which a significant rise in PCWP > 15 mmHg, was seen in 47% of the patients, the majority of whom continued to experience rising PCWP, above > 25 mmHg, during exercise. Well-defined cutoff values for PCWP during PPL and exercise are not well established. However, according to recent guidelines [18] PCWP > 15 mmHg at rest is recommended as a marker for raised resting LVFPs. Furthermore, with PLL and submaximal exercise, the majority of patients with normal NT-proBNP did not exceed PCWP of 15 mmHg and 25 mmHg, respectively [19]. In addition, the close relationship between the values obtained during PPL and exercise, illustrates the significant accuracy of the PLL maneuvers ability to predict hemodynamics outcomes and effects during exercise. A number of explanations for this relationship need to be discussed, in particular the effect of venous return and ventricular–arterial coupling, commonly known to be disturbed in HF [20, 21].

With PLL, and during exercise, the venous return to the heart increases, which, in the presence of a stiff LV cavity, is coupled to an increase in LA pressures hence a subsequent rise in PCWP [8, 22–24]. However, exercise also influences afterload. Significant hemodynamic differences among patients with or without elevated NT-proBNP become evident when challenged, even though NT-proBNP levels are raised within the entire eBNP group. It has been reported that patients with elevated NT-proBNP levels and normal hemodynamic response may be attributed to the protective properties of increased BNP levels. However, in our patients we found resting NT-proBNP to be accurate in identifying abnormal PCWP increase, both during PLL and exercise.

The RV afterload is the total pulmonary vascular resistance (TPVR), constituted by the sum of the transpulmonary gradient and PCWP, divided by CO. An increased TPVR, regardless of resting or stress conditions, suggests underlying disease within the pulmonary vascular or post capillary system, with an exercise mPAP/CO ratio of > 3 WU predicting exercise pulmonary hypertension [25, 26]. Current definition [27] of exercise pulmonary hypertension also includes exercise-mPAP > 30 mmHg criterion.

Since PVR is fixed, or decreases during exercise, rapidly elevated left-sided filling pressures should be considered if mPAP/CO increases during stress. Using the mPAP/CO changes during exercise in our analysis showed that PVR did not have any potential influence on the pressure and flow alterations found in this study, thus leaving increased left ventricular FPs as the main explanation of mPAP/CO increase. Recently [24] PCWP/CO during exercise has been found to predict exercise capacity and HF outcome; however, it is known to be accurately assessed by echocardiography.

In this study, other factors, i.e., age and atrial fibrillation were robust measures highly indicative of elevated LVFPs during exercise, as well as weight in indicating raised high PCWP at rest. An important finding in our study is that the use of PLL and/or supine bicycling in patients with NT-proBNP of 125 ng/l or more, better stratifies patients, while NT-proBNP < 125 ng/l remains a robust marker of normal PCWP, therefore, making heart failure highly unlikely. This is in accordance with previous trials of HFpEF which consistently show that NT-proBNP is the most powerful prognostic marker [17].

### Clinical implications

In patients with eBNP, resting RHC measurements showing normal PCWP should not be taken as a complete comprehensive examination, and consequently the same limitation applies for echocardiography. In case of normal findings at rest, some sort of stress test has to be undertaken to grade HF [15, 28, 29]. Our findings support the current guidelines which highlight the important use of stress echocardiography in the routine management of HF patients [30, 31]. They propose PLL as a complementary simple investigation, which could be used in all patients with exertional dyspnea, as means for assessing LVFPs using echocardiography, in an outpatient setting. The use of echocardiography in PLL remains to be tested and the most accurate markers of PCWP identified before it becomes clinically applicable. The changes in mPAP/CO in HF patients might also be of specific interest as it can be assessed by non-invasive Doppler echocardiography.

## Limitations

This is a retrospective, non-randomised, single-center study. Both patient groups were taking medications, but more commonly in the patient group with elevated NT-proBNP, which could influence the measurements. The nBNP patients were younger than eBNP patients, a difference that may influence the group comparison. Even though the nBNP group had normal NT-proBNP levels, these patients cannot be considered to represent a “healthy population”, despite having normal hemodynamics. We did not have detailed echocardiographic results for these patients prior to the RHC. Patients were only encouraged to perform submaximal exercise and most did not develop severe symptoms. The cutoff for abnormality values from NT-proBNP ( > 125 ng/l), PCWP ( > 15/25 mmHg) and mPAP ( > 25/30 mmHg) at rest and exercise can be argued. However, we relied mainly on the recommendations published [18, 32].

## Conclusion

The addition of PLL during RHC unmasked disturbed LV physiology and raised filling pressures in most patients presenting with eBNP. PLL had a sensitivity of 91% and specificity 92% for identifying significantly raised PCWP with exercise, which is the likely cause of unexplained dyspnea in these patients. Therefore, the use of PLL and exercise could be proposed as complimentary test for HF patients. Finally, using changes in mPAP/CO during exercise could also be proposed as a useful method for detecting abnormal filling pressures with stress.

## Electronic supplementary material

Below is the link to the electronic supplementary material.

Supplementary file1 (PDF 99 kb)
