# Supplementary material for: Passive leg-lifting in heart failure patients predicts exercise-induced rise in left ventricular filling pressures
Source: Clin Res Cardiol. 2019 Jul 31;109(4):498–507. doi: 10.1007/s00392-019-01531-w (PMC7098926; doi:10.1007/s00392-019-01531-w)
Supplement: Supplementary file 1 — Supplementary file1 (PDF 99 kb) [file 392_2019_1531_MOESM1_ESM.pdf]

Supplemental Materials.

Table 3

Groups classified according to PCWP at rest and exercise

	PCWPn(rest) - PCWPn(ex) (62)	PCWPn (rest) - PCWPe (ex) (12)	PCWPe(rest) - PCWPe (ex) (11)	p-value
Age, years	57 (45-68)	70 (69-76) ¤	67 (63-73) ƫ	<0.001
Female	31	7	4	0.566
ACE inhibitors	33	8	8	0.383
Betablockers	21	8 ¤	8 ƫ	0.012
Diuretics	14	5	5	0.161
Calcium channel blockers	11	3	7 ƫ	0.005
Atrial fibrillation or flutter	7	7 ¤	5 ƫ	<0.001
IHD	7	1	2	0.746
SHT	21	8 ¤	8 ƫ	0.019
DM	3	3 ¤	3 ƫ	0.018
NT-pro BNP, ng/L	93 (51-266)	486 (198-2679) ¤	880 (281-1785) ƫ	0.034
Creatinine, micro mol/L	78 (63-94)	93 (66-129)	80 (68-92)	0.192
GFR, ml/min	80 (52-100)	61 (44-80)	70 (50-83)	0.124
Systolic blood pressure, mmHg	125 (120-140)	140 (113-150)	147 (125-150)	0.077
Workload, Watt	50 (40-70)	40 (30-50) ¤	50 (40-50)	0.024
Heart rate, rest, bpm	70 (60-80)	63 (55-75)	68 (58-76)	0.494
Height, cm	174 (167-180)	176 (162-180)	174 (167-178)	0.999
Weight, kg	79 (71-88)	82 (76-91)	94 (90-100) ᴪ ƫ	0.001

Kruskal-Wallis H assessment of differences in continuous variables between groups. Cochran–Mantel–Haenszel test was used for analysis of categorical data. ¤ p<0.05 compared with PCWP Low-Low (Mann Whitney U), ƫ p<0.05 compared with PCWP Low-Low (Mann Whitney U), ᴪ p<0.05 compared with PCWP Low-High (Mann Whitney U). Out of the patients with PCWP Low-Low, one patient had Thiazide treatment instead of loop diuretics and one patient in the High at rest group was treated with both Thiazide and Loop diuretics. Abbreviations: IHD=ischemic heart disease, SHT=systemic hypertension, DM=diabetes mellitus, BNP=brain natriuretic peptides, GFR=glomerular filtration rate, n=normal, e=elevated.

Table 4

Groups classified according to PCWP at rest and exercise

	nPCWP(rest) - nPCWP(ex) (62)	nPCWP (rest) - ePCWP (ex) (12)	ePCWP(rest) - ePCWP (ex) (11)	p-value
**Resting data RHC**				
mPAP, mmHg	15 (13-18)	21 (18-24) ¤	28 (26-32) † Ψ	<0.001
CO, l/min	5.4 (4.6-6.4)	5.8 (4.2-7.1)	7.0 (5.6-8.0) †	0.01
PCWP/CO, mmHg/l/min	1.4 (1.0-1.8)	1.7 (1.0-2.5)	2.6 (2.2-3.2) † Ψ	<0.001
mPAP/CO, mmHg/l/min	2.8 (2.2-3.4)	3.9 (3.0-6.0) ¤	4.3 (3.7-5.1) †	<0.001
PCWP, mmHg	8 (6-10)	10 (6-12)	17 (16-18) † Ψ	<0.001
PVR, WU	1.2 (0.9-1.9)	2.1 (1.4-3.2)	1.6 (1.2-2.4)	0.01
TPG,mmHg	7 (6-10)	10.0 (7-16)	12 (9-15) †	0.001
**PLL data RHC**				
mPAP, mmHG	20 (17-24)	30 (26-34) ¤	38 (32-41) † Ψ	<0.001
PCWP, mmHg	11 (9-13)	18 (16-19) ¤	23 (21-26) † Ψ	<0.001
TPG, mmHg	9 (6-12)	12 (10-17) ¤	12 (9-17) †	<0.001
**Exercise data RHC**				
mPAP, mmHg	29 (23-36)	46 (40-53) ¤	52 (48-57) †	<0.001
CO, l/min	10.7 (9.5-12.3)	8.9 (7.4-10.0) ¤	9.6 (8.2-11.2)	0.002
PCWP/CO, mmHg/l/min	1.6 (1.1-2.0)	3.5 (3.0-4.6) ¤	3.4 (3.0-4.1) †	<0.001
mPAP/CO, Hg/ml/min	2.8 (2.0-3.5)	5.4 (4.6-7.5) ¤	5.8 (4.5-7.0) †	<0.001
PCWP, mmHg	18 (13-21)	30 (27-35) ¤	33 (28-39) †	<0.001
PVR, WU	1.1 (0.7-1.6)	1.9 (1.2-2.5) ¤	2.4 (1.6-2.7) †	0.005
TPG,mmHg	11 (8-16)	13 (10-22)	20 (15-27) †	0.10

Kruskal-Wallis H assessment of differences between groups.

¤ p<0.05 compared with Low-Low (Mann Whitney U), † p<0.05 compared with Low-Low (Mann Whitney U), Ψ p<0.05 compared with Low-High (Mann Whitney U)

There was no difference in PCWP or PCWP/CO between Low-Low and Low-High PCWP at rest, however PCWP was significantly higher at both PLL and exercise, also PCWP/CO was significantly higher at exercise compared to rest. Interestingly there were no statistical differences in hemodynamics between Low-High and High at rest, during exercise.

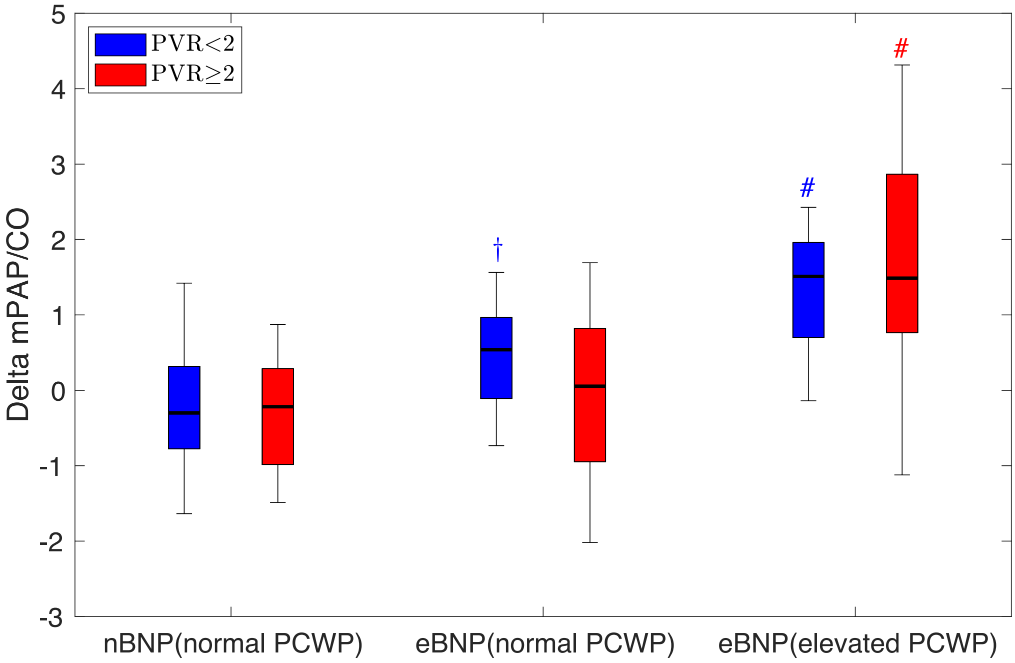

Fig. 7 Delta (exercise-rest) mPAP/CO (mmHg/L/min) in nBNP (left), eBNP with normal PCWP at exercise (middle), and in eBNP with elevated PCWP at exercise (right). Right red boxplots are patients with PVR ≥ 2WU and leftr blue < 2WU. Note that the delta mPAP/CO was not related to the level of PVR. † $p<0.05$ eBNP with normal PCWP vs nBNP, within the same PVR groups.
# $p<0.05$ eBNP with elevated PCWP vs normal PCWP, within the same PVR groups
Boxes show median and IQR, whiskers show range.
